# The left atrial appendage closure by surgery 2 trial: statistical analysis plan for a randomized multicenter trial exploring if the closure of the left atrial appendage during open-heart surgery reduces stroke irrespective of patients’ stroke risk and preoperative atrial fibrillation status

**DOI:** 10.1186/s13063-024-08122-9

**Published:** 2024-05-14

**Authors:** Christoffer L. Madsen, Jesper Park-Hansen, Rakin Hadad, Anders M. Greve, Helena Domínguez

**Affiliations:** 1 Department of Cardiology, Copenhagen University Hospital—Bispebjerg and Frederiksberg, Nordre Fasanvej 57, 2000 Frederiksberg, Copenhagen, Denmark; 2 Department of Biomedical Science, University of Copenhagen, Copenhagen, Denmark; 3 Department of Clinical Biochemistry, Copenhagen University Hospital—Rigshospitalet, Copenhagen, Denmark

**Keywords:** Left atrial appendage occlusion, Left atrial appendage exclusion, Open-heart surgery, Stroke, Transient ischemic attack, Atrial fibrillation, Statistical analysis plan

## Abstract

**Background:**

Surgical left atrial appendage (LAA) closure concomitant to open-heart surgery prevents thromboembolism in high-risk patients. Nevertheless, high-level evidence does not exist for LAA closure performed in patients with any CHA_2_DS_2_-VASc score and preoperative atrial fibrillation or flutter (AF) status—the current trial attempts to provide such evidence.

**Methods:**

The study is designed as a randomized, open-label, blinded outcome assessor, multicenter trial of adult patients undergoing first-time elective open-heart surgery. Patients with and without AF and any CHA_2_DS_2_-VASc score will be enrolled. The primary exclusion criteria are planned LAA closure, planned AF ablation, or ongoing endocarditis. Before randomization, a three-step stratification process will sort patients by site, surgery type, and preoperative or expected oral anticoagulation treatment. Patients will undergo balanced randomization (1:1) to LAA closure on top of the planned cardiac surgery or standard care. Block sizes vary from 8 to 16. Neurologists blinded to randomization will adjudicate the primary outcome of stroke, including transient ischemic attack (TIA). The secondary outcomes include a composite outcome of stroke, including TIA, and silent cerebral infarcts, an outcome of ischemic stroke, including TIA, and a composite outcome of stroke and all-cause mortality. LAA closure is expected to provide a 60% relative risk reduction. In total, 1500 patients will be randomized and followed for 2 years.

**Discussion:**

The trial is expected to help form future guidelines within surgical LAA closure. This statistical analysis plan ensures transparency of analyses and limits potential reporting biases.

**Trial registration:**

Clinicaltrials.gov, NCT03724318. Registered 26 October 2018, https://clinicaltrials.gov/study/NCT03724318.

**Protocol version:**

10.1016/j.ahj.2023.06.003.

**Supplementary Information:**

The online version contains supplementary material available at 10.1186/s13063-024-08122-9. The SAP checklist is supplied in the supplementary material.

## Introduction

### Background and rationale

Patients undergoing open-heart surgery generally have many comorbidities and a high prevalence of atrial fibrillation (AF), yielding an elevated risk of stroke compared to the background population [1–5]. The left atrial appendage (LAA) is a highly trabeculated predilection point for thrombus formation, especially during AF, where there is poor atrial contraction and reduced blood flow in the atria [6–8]. The thrombi may dislodge and follow the arterial bloodstream to the brain, causing a stroke [6–8]. In 2018, a randomized clinical trial showed that surgical closure of the LAA resulted in a lower incidence of combined stroke, transient ischemic attack (TIA), and silent cerebral infarction (SCI) compared to leaving the LAA open [9]. Although a trend was noted, the trial was underpowered to answer whether surgical LAA closure protects against stroke and TIA [9]. Other studies suggest that LAA closure performed in addition to open-heart surgery is safe and can reduce the incidence of thromboembolism [10–12]. Accordingly, current guidelines declare that surgical LAA closure may be considered in patients with AF undergoing open-heart surgery (Class IIb) [10–15]. Recently, the largest randomized clinical trial to date reported that surgical LAA closure reduced the risk of systemic thromboembolism and stroke in patients with AF and a high risk of thromboembolism (CHA_2_DS_2_-VASc ≥ 2) [13]. Nevertheless, whether LAA closure is safe and advantageous for patients without AF and a low á priori stroke risk remains unknown [6, 16, 17]. The rationale of the LAACS-2 trial is to provide such evidence. Accordingly, the LAACS-2 trial will evaluate if prophylactic LAA closure as a standard add-on to open-heart surgery is safe and can reduce the risk of stroke and TIA events. The recommended LAA closure is by an epicardial device, with open-heart surgery without LAA closure as the comparator.

### Objectives

#### Primary objective

To test the hypothesis that closure of the LAA concomitantly with elective open-heart surgery can reduce the incidence of stroke compared to leaving the LAA open, regardless of previous AF diagnosis and CHA_2_DS_2_-VASc score.

#### Secondary objectives

To evaluate if the incidence of combined stroke, TIA, and SCI may be reduced by closing the LAA in the same population as above.

To evaluate if the incidence of combined ischemic stroke, TIA, and SCI may be reduced by closing the LAA in the same population as above.

To evaluate if all-cause mortality and stroke incidence can be reduced by closing the LAA in the same population as above.

To evaluate the safety of LAA closure performed concomitantly with elective open-heart surgery in the same population as above.

## Study methods

### Trial design

A randomized, multicenter trial of LAA closure performed during surgery in addition to planned open-heart surgery. The comparator is leaving the LAA open. The preferred method of LAA closure is by an epicardial device (AtriClip®, AtriCure Inc., West Chester, Ohio, USA). Alternatively, a stapler may amputate the LAA at the surgeon’s discretion. After the surgical intervention, there are no limitations in anticoagulant use, heart rate-limiting, or rhythm-controlling treatment, including cardioversion attempts. Adherence to current clinical guidelines is recommended.

### Framework

The hypothesis testing framework is that of a superiority trial. The hypothesis being tested is that LAA closure is superior to leaving the LAA open when concerning the primary outcome. Moreover, that there is no difference between LAA closure and leaving the LAA open when considering safety outcomes. No power calculations have been performed for the safety outcomes.

### Randomization and stratification

Before randomization, a three-step stratification is performed. The first step is “site,” the second is “surgery type,” and the third is “use of oral anticoagulant (OAC)” (see the table below).

**Table:** 

Site	Aarhus University Hospital, Denmark
	Copenhagen University Hospital—Rigshospitalet, Denmark
	Sahlgrenska University Hospital, Sweden
	Vall d'Hebron University Hospital, Spain
Surgery type	Isolated coronary artery bypass grafting (CABG)
	Mitral valve surgery ± other surgery
	Non-mitral valve surgery ± CABG
OAC	Prior or expected use of vitamin K or non-vitamin K OAC or patient planned for mechanical valve surgery
	No OAC before surgery or expected post-surgery, and a patient planned for mechanical valve surgery

For each stratum, patients will be randomized 1:1 to LAA closure or leaving the LAA open. The Research Electronic Data Capture (REDCap) randomization module will be used to assign treatment allocation. A randomization sequence was created and uploaded by the Steering Committee before trial initiation. The block sizes vary from 8 to 16. After the initiation of the trial, no changes will be made to the sequence. On the day of surgery, the site’s study investigator will randomize patients in the REDCap randomization module. After assigning treatment allocation, no changes will be allowed in the randomization module for the specific research subject. The Steering Committee does not have access to the randomization module after trial initiation, while the sites do not have access to the randomization sequence.

Data on treatment allocation is available to the local healthcare professional. Surgeons are allowed to reveal treatment allocation if requested by the patient.

### Sample size

Based on previous studies, estimated event rates are 1.2% vs. 3.0% per year for patients with and without LAA closure, respectively [8, 9, 11, 16, 18, 19]. A total of 1302 patients need to be included if they are randomized 1:1, followed for 2 years, and a significance level of 0.05 and 90% power is to be achieved. With these numbers, 16 primary events are expected for patients with LAA closure and 39 for patients with an open LAA. The trial will enroll 1500 patients to account for deviations in treatment allocation and death. Please see the trial protocol for more details [20].

### Statistical interim analyses, safety, and stopping guidance

No interim analyses will be conducted. An analysis and evaluation of adverse events will be performed by the trial Data Safety Monitoring Board annually. The Data Safety Monitoring Board will recommend that the Steering Committee discontinue the trial if there is reasonable concern that LAA closure increases the risk of adverse events.

### Timing of the final analysis

The final analysis is scheduled for when 55 primary events have occurred, which is anticipated once the last patient recruited has undergone 2 years of follow-up (please see section on “sample size”). The Steering Committee will decide whether to shorten follow-up if 55 primary events have occurred before 2 years of follow-up for all patients or before 1500 patients have been enrolled. Contrarily, the Steering Committee may opt to extend follow-up if fewer than 55 events have occurred once the last enrolled patient has 2 years of follow-up.

### Timing of outcome assessments

The primary and secondary outcomes will be assessed at discharge, 3 months after surgery, and then annually until the end of follow-up.

Safety outcomes will be assessed 30 days after index surgery and, subsequently, at the same time intervals as the abovementioned outcomes.

## Statistical principles

### Confidence intervals and *P* values

A significance level of 5% and a confidence interval of 95% will be applied. No multiplicity adjustment is planned; consequently, findings in secondary outcomes are considered exploratory. Only two-sided significance tests will be used.

### Adherence and protocol deviations

Adherence to the treatment assignment denotes whether patients follow the assigned randomization (LAA closure vs. leaving the LAA open). Any patient not following the assigned randomization will be considered a protocol violation. Adherence to the intervention will be presented as a proportion relative to the intended allocation. If LAA closure is performed on a clinical indication during follow-up, the patient’s follow-up will be halted at the moment of closure. Such cases will be reported separately.

### Analyses of the populations

All outcomes will be analyzed as intention-to-treat (ITT) and per-protocol (PP). In the ITT analyses, the assigned randomization is the basis of the segregation of treatment groups. Only data from patients with LAA status congruent with randomization will be included in the PP analyses.

## Trial population

### Screening data

Patients referred for elective open-heart surgery at each site will be screened for eligibility. Sites register the total number of screened patients, whether in- and exclusion criteria have been met, and whether exclusion is due to patient dissent to study participation or planned LAA closure. Summed screening data will be presented in the main publication.

### Eligibility

#### Inclusion criteria

- Patients ≥ 18 years
- Elective open-heart surgery (CABG, valve, or combined)
- Signed informed consent

#### Exclusion criteria

- Prior open-heart surgery
- Current endocarditis
- Planned closure of the LAA
- Planned ablation for AF
- Follow-up impossible

### Recruitment

Recruitment data will be presented in a Consolidated Standards of Reporting Trials (CONSORT) flow diagram. The diagram will include data on the estimated number of patients assessed for eligibility per site and the number of patients excluded, including reasons. Furthermore, the number of patients randomized and the two allocation groups (including data on whether patients received the allocated intervention as intended) will be shown. Subsequently, any loss to follow-up and patients excluded for other reasons will be displayed, leaving the final dataset available for analysis.

### Withdrawal/follow-up

Patients may retract their consent at any time point during follow-up. The intervention cannot be undone; therefore, retraction of consent after surgery entails that follow-up data will no longer be gathered. If consent is retracted, the date will be registered, and data will be included in the analysis until this date if accepted by the patient. Alternatively, all data will be deleted if requested by the patient. In this case, data will not be included in the analysis. The number of patients retracting consent and their reasons will be presented in the CONSORT flow diagram.

### Baseline patient characteristics

The following baseline patient characteristics will be presented:

I. Demographics: age and sex
II. Measurements:
  ◦ Optional: height, weight, blood pressure, left ventricular ejection fraction, and estimated glomerular filtration rate
III. Comorbidities: prior stroke, TIA, AF, or atrial flutter
  ◦ Optional: ischemic heart disease, peripheral artery disease, cardiac valve disease, congestive heart failure, pacemaker or implantable cardioverter defibrillator implantation, hypertension, chronic obstructive/restrictive pulmonary disease, diabetes mellitus, chronic kidney disease, autoimmune disease, active cancer
IV. Medications: any heart rhythm or frequency modulators, anticoagulation, or antiplatelet therapy
V. Prior brain scans: any brain scan performed before study enrolment, including findings

Continuous variables will be summarized as mean (standard deviation) or median (interquartile range), while categorical variables are summarized as frequencies (percentages).

## Analysis

### Outcome definitions

#### Primary outcome

**Table:** 

Outcome	Definition	Time frame
Stroke and TIA	The occurrence of stroke, including TIA. Both are considered clinical diagnoses constituted by acute focal or global neurological symptoms brought on by thromboembolism or hemorrhage in the brain. The event is defined as a TIA if symptoms last less than 24 h. If patients with a stroke diagnosis receive thrombolytic treatment leading to complete symptom remission, the event is considered a stroke. If patients are diagnosed with amaurosis fugax, the event will be regarded as a TIA. If patients are diagnosed with retinal artery occlusion, the event will be considered a stroke. The stroke subtypes will not be discerned in the primary outcome analyses, i.e., both ischemic and hemorrhagic types are included. The first adjudicated primary outcome is included, while later occurring events are disregarded.	2 years from index surgery

#### Secondary outcomes

**Table:** 

Outcome	Definition	Time frame
Stroke, TIA, and SCI (composite)	Composite outcomes of stroke, including TIA and SCI. Stroke and TIA are defined as for the primary outcome and are included regardless of the results of computed tomography (CT) or magnetic resonance imaging (MRI) scans. SCI entails a fresh lesion on a CT or MRI scan without concomitant symptoms or a new-onset lesion of an older date in patients with recurring scans. The stroke subtypes will not be discerned in the primary outcome analyses, i.e., both ischemic and hemorrhagic types are included.	2 years from index surgery
Ischemic stroke, including TIA	The diagnosis of stroke or TIA is defined in the primary outcome, but where CT or MRI scan(s) show ischemic lesion congruent with symptoms.	2 years from index surgery
All-cause mortality and stroke (composite)	Death of any cause or the diagnosis of stroke. The diagnosis of stroke is defined as the primary outcome. There is no discerning between stroke subtypes. TIA is not included in this outcome.	2 years from index surgery

The outcomes will be analyzed in the order they are reported

Neurological outcomes will be adjudicated by two neurologists. If a neurological event occurs, the site’s study investigators will draw a report from the patient’s medical chart. The report includes data on patient symptoms, clinical and paraclinical examinations performed, cerebral CT or MRI scan descriptions (as described by neurological radiologists), and any medication initiated during admission. The reports will be anonymized, and the neurologists will be blinded to study allocation. The events will be assigned by consensus of the two neurologists if there are discrepancies in the adjudications.

Stroke subtypes will be differentiated by cerebral CT or MRI scans showing lesions congruent with the symptoms. In the case of hemorrhagic transformation of an ischemic stroke, the event will be considered an ischemic stroke. Moreover, retinal artery occlusions will also be considered an ischemic stroke event.

SCI is a lesion on cerebral CT or MRI scans without neurological symptoms. The SCI may either be fresh ischemic, i.e., showing hyper-intensity on diffusion-weighted imaging (DWI) and apparent diffusion coefficient (ADC) hypo-intensity, or a new-onset non-fresh lesion in a patient with one or more previous scans.

No study-specific cerebral CT or MRI scans will be performed; accordingly, only scans performed in a clinical setting will be considered.

#### Other outcomes

**Table:** 

Outcome	Definition	Time frame
Systemic embolism	A clinical diagnosis of an acute non-cerebral artery occlusion	2 years from index surgery
AF occurrence or recurrence	The recurrence or occurrence of AF during follow-up for patients with sinus rhythm at discharge after index surgery	2 years from index surgery
All-cause mortality	Death from any cause	2 years from index surgery

New-onset AF occurring from index surgery until discharge will be considered “postoperative AF”. If patients are discharged in sinus rhythm and have an occurrence or recurrence of AF within 3 months from index surgery, the episode will be defined as an “early” AF occurrence or recurrence, while AF occurrence after 3 months will be defined as “late”. Only the first incidence of postoperative AF and the first incidence of occurrence or recurrence of AF will be registered. No study-specific continuous cardiac rhythm monitoring will be performed during admission; hence, the evaluation will be based on AF detection in the clinical setting and rely on registration in the electronic medical chart. Any available electrocardiogram (ECG) will be used to verify AF. Continuous cardiac rhythm monitoring after discharge is planned at selected sites for a subgroup of patients with postoperative AF discharged in sinus rhythm and without recurrence of AF in the subsequent years. If more than 30 s of AF is seen on the monitoring or a 12-lead ECG, patients will be considered to have AF recurrence.

### Analysis methods

The respective investigators gather data from each site, and primary and secondary analyses will be performed centrally.

The primary analyses rely on time-to-event (survival) statistics.

The possible events are as follows:

I. Primary outcome occurrence
II. Death
III. Withdrawal of consent
IV. Last contact (loss to follow-up)
V. Trial end*

*The time frame is at least 2 years from index surgery; thus, follow-up continues for all patients until the last enrolled patient has been followed for 2 years.

The time between randomization and event (I–V) is the exposure time.

The primary analyses will be based on the ITT principle, i.e., it includes all randomized patients with an available baseline assessment independently of the performed surgery.

PP analyses will serve as sensitivity analyses.

#### Analyses of the primary outcome

Cumulative incidence (Aalen-Johansen) plots will display results of the primary outcome with death as a competing risk. Gray’s test for differences between cumulative incidence rates will be used to assess statistical significance.

As the primary outcome will be examined in a competing risk setting, cause-specific Cox regression is used to quantify the difference in risk between allocation groups. The main covariate is randomization (LAA closure vs. open LAA). The analysis will be adjusted for the stratification factors before randomization, i.e., site, surgery type, and OAC therapy. The results will be presented as hazard ratio (HR) with 95% confidence intervals.

The null hypothesis is that there is no difference between the cumulative incidence rates of the two treatment groups.

The decision rule for the hazard ratio is as follows:

VI. Superiority of LAA closure will be accepted if the estimated HR is < 1 and the 95% confidence interval (CI) does not include 1
VII. Superiority of open LAA will be accepted if the estimated HR is > 1 and the 95% CI does not include 1
VIII. No superiority will be accepted if the 95% CI contains 1

Proportional hazard assumptions for the Cox regression models will be tested by interaction terms with time on study, weighted Schoenfeld, and cumulative Martingale residuals. If non-proportionality is detected for covariables used for adjustment (site, surgery type, and OAC therapy), these factors will be included as stratification variables. If non-proportionality is detected for the primary exposure (randomization), time-varying hazard ratios with 95% confidence intervals will be used to assess the differential effect of randomization as a function of time-on study.

#### Analyses of the key secondary outcomes

The secondary outcomes will be analyzed as described for the primary outcome. The third secondary outcome of all-cause mortality and stroke will use the Kaplan–Meier estimator to visualize survival times. The comparison is based on the Cox proportional-hazard model with two-sided 95% CIs. All secondary outcome analyses will be considered exploratory.

#### Sensitivity analyses

Sensitivity analyses based on PP allocation will be used to test the robustness of the primary analyses. The statistical analyses are equivalent to those described above. Moreover, a complete-case analysis will be performed.

#### Subgroup analyses

Subgroup analyses will investigate the interaction of outcomes and key stroke risk factors. Predefined analyses are as follows:

I. Prior stroke, including TIA
II. Prior AF
III. Recurrent AF
IV. Pre-operative CHA_2_DS_2_-VASc score
V. Preoperative and postoperative OAC
VI. Preoperative and postoperative antiplatelet therapy

The interaction terms will be added to the Cox models described in the primary and secondary outcome analyses above. A significant subgroup effect will be considered if the *p*-value ≤ 0.05. Each interaction term’s HR and 95% CI will be presented in a forest plot.

### Missing data

No missing data is anticipated for the primary analyses (time-to-event statistics) of the primary and secondary outcomes. In secondary (sensitivity) analyses, missing data will be handled by multiple imputation with the fully conditional specification method. If patients retract their consent after randomization, follow-up data gathering stops at the retraction date. The follow-up data will be included in the analyses unless the patients request that their data is deleted.

### Additional analysis

#### Long-term follow-up studies

Intended long-term follow-up studies entail a review of patients’ health records for new primary and secondary events after 5 and 10 years, as calculated from the last enrolled patient’s randomization date.

#### Planned substudies

The substudies will be analyzed and published separately from the main study publications and are briefly described.

- ECG substudy: The aim is to improve the prediction of (1) postoperative AF and (2) postoperative stroke, including TIA, using AI-based ECG and clinical data. A baseline ECG has been obtained for all patients.
- wavECG substudy: the aim is to identify the presence of an arrhythmogenic substrate using wavECG (Myovista electrocardiogram, HeartSciences, Southlake, Texas, USA) data along with clinical data and biological biomarkers such as blood and tissue samples. A baseline wavECG has been obtained for a subgroup of patients, expected *n* = 200.
- The imaging substudy: the aim is to evaluate LAA closure efficacy. A subgroup of ten consecutive patients at each site will be examined by transesophageal echocardiography, cardiac CT, or MRI, as applicable at each site. In addition, patients with LAA closure developing ischemic stroke, including TIA, are recommended an examination of their LAA. An estimated 50 patients are included.
- Holter substudy: the aim is to evaluate the burden of asymptomatic AF recurrence. Patients with new-onset AF after index surgery and sinus rhythm at discharge may be invited for ambulatory continuous cardiac rhythm monitoring approaching the end of follow-up. Patients with an AF episode during follow-up will not be eligible for monitoring. It is estimated that 100 patients will be monitored.

### Harms

The epicardial LAA closure procedures that will be used in this trial are deemed safe and are commercially available. Nonetheless, the following safety outcomes will be systematically recorded and used for annual safety assessments.

**Table:** 

Item	Time frame
Bleeding (BARC 3a, 3b, 4)^*^	Within 30 days from index surgery
Infection	
Redo cardiac surgery^#^	
Re-hospitalization due to • Pericardial effusion • Pleural effusion • Pericardial infection • Acute decompensated heart failure • Bleeding (BARC 2–4)^*^	30 days from index surgery until the end of follow-up

^*^The Bleeding Academic Research Consortium (BARC) criteria define bleeding events [21]. ^#^Including redo surgery due to device failure or injury caused by device placement

All randomized patients undergoing index surgery will be included in the safety analysis data set. The number and frequencies of the adverse events in each allocation group will be presented in tables alongside descriptive statistics.

### Statistical software

The most recent version of RStudio (Boston, MA, USA) will be used for statistical analysis.

### Supplementary Information

**Supplementary Material 1.**

## Abbreviations

AF: Atrial fibrillation or flutter
BARC: Bleeding Academic Research Consortium
CABG: Coronary artery bypass grafting
CI: Confidence interval
CONSORT: Consolidated Standards of Reporting Trials
CT: Computed tomography
ECG: Electrocardiogram
HR: Hazard ratio
ITT: Intention-to-treat
LAA: Left atrial appendage
LAACS: Left atrial appendage closure by surgery
MRI: Magnetic resonance imaging
OAC: Oral anticoagulation
PP: Per-protocol
SCI: Silent cerebral infarction
TIA: Transient ischemic attack
