# Supplementary material for: The left atrial appendage closure by surgery 2 trial: statistical analysis plan for a randomized multicenter trial exploring if the closure of the left atrial appendage during open-heart surgery reduces stroke irrespective of patients’ stroke risk and preoperative atrial fibrillation status
Source: Trials. 2024 May 14;25:317. doi: 10.1186/s13063-024-08122-9 (PMC11092018; doi:10.1186/s13063-024-08122-9)
Supplement: Supplementary file 1 — Supplementary Material 1. [file 13063_2024_8122_MOESM1_ESM.pdf]

Statistical Analysis Plan (SAP) Checklist v 1.0 2019

Section/Item	Index	Description	Reported on page #
**Section 1: Administrative information**			
Trial and Trial registration	1a	Descriptive title that matches the protocol, with SAP either as a forerunner or subtitle, and trial acronym (if applicable)	1
	1b	Trial registration number	2
SAP Version	2	SAP version number with dates	1
Protocol Version	3	Reference to version of protocol being used	3
SAP revisions	4a	SAP revision history	N/A
	4b	Justification for each SAP revision	N/A
	4c	Timing of SAP revisions in relation to interim analyses, etc.	N/A
Roles and responsibility	5	Names, affiliations, and roles of SAP contributors	1 & 20
Signatures of:	6a	Person writing the SAP	-
	6b	Senior statistician responsible	-
	6c	Chief investigator/clinical lead	-
**Section 2: Introduction**			
Background and rationale	7	Synopsis of trial background and rationale including a brief description of research question and brief justification for undertaking the trial	3
Objectives	8	Description of specific objectives or hypotheses	4
**Section 3: Study Methods**			
Trial design	9	Brief description of trial design including type of trial (e.g., parallel group, multi-arm, crossover, factorial) and allocation ratio and may include brief description of interventions	5
Randomization	10	Randomization details, e.g., whether any minimization or stratification occurred (including stratifying factors used or the location of that information if it is not held within the SAP)	5
Sample size	11	Full sample size calculation or reference to sample size calculation in protocol (instead of replication in SAP)	6
Framework	12	Superiority, equivalence, or noninferiority hypothesis testing framework, including which comparisons will be presented on this basis	5
Statistical interim analysis and stopping guidance	13a	Information on interim analyses specifying what interim analyses will be carried out and listing of time points	6
	13b	Any planned adjustment of the significance level due to interim analysis	N/A
	13c	Details of guidelines for stopping the trial early	6

Timing of final analysis	14	Timing of final analysis, e.g., all outcomes analysed collectively or timing stratified by planned length of follow-up	7
Timing of outcome assessments	15	Time points at which the outcomes are measured including visit “windows”	7
**Section 4: Statistical Principals**			
Confidence intervals and *P* values	16	Level of statistical significance	7
	17	Description and rationale for any adjustment for multiplicity and, if so, detailing how the type 1 error is to be controlled	N/A
	18	Confidence intervals to be reported	7
Adherence and Protocol deviations	19a	Definition of adherence to the intervention and how this is assessed including extent of exposure	7-8
	19b	Description of how adherence to the intervention will be presented	8
	19c	Definition of protocol deviations for the trial	7-8
	19d	Description of which protocol deviations will be summarized	8
Analysis populations	20	Definition of analysis populations, e.g., intention to treat, per protocol, complete case, safety	8
**Section 5: Trial Population**			
Screening data	21	Reporting of screening data (if collected) to describe representativeness of trial sample	8
Eligibility	22	Summary of eligibility criteria	8-9
Recruitment	23	Information to be included in the CONSORT flow diagram	9
Withdrawal/ Follow-up	24a	Level of withdrawal, e.g., from intervention and/or from follow-up	9
	24b	Timing of withdrawal/lost to follow-up data	9
	24c	Reasons and details of how withdrawal/lost to follow-up data will be presented	9
Baseline patient characteristics	25a	List of baseline characteristics to be summarized	10
	25b	Details of how baseline characteristics will be descriptively summarized	10
**Section 6: Analysis**			
Outcome definitions		List and describe each primary and secondary outcome including details of:	
	26a	Specification of outcomes and timings. If applicable include the order of importance of primary or key secondary end points (e.g., order in which they will be tested)	11
	26b	Specific measurement and units (e.g., glucose control, hbA1c [mmol/mol or %])	N/A
	26c	Any calculation or transformation used to derive the outcome (e.g., change from baseline, QoL score, Time to event, logarithm, etc.)	11
Analysis methods	27a	What analysis method will be used and how the treatment effects will be presented	14-16

	27b	Any adjustment for covariates	15-16
	27c	Methods used for assumptions to be checked for statistical methods	16
	27d	Details of alternative methods to be used if distributional assumptions do not hold, e.g., normality, proportional hazards, etc.	N/A
	27e	Any planned sensitivity analyses for each outcome where applicable	16
	27f	Any planned subgroup analyses for each outcome including how subgroups are defined	16-17
Missing data	28	Reporting and assumptions/statistical methods to handle missing data (e.g., multiple imputation)	17
Additional analyses	29	Details of any additional statistical analyses required, e.g., complier-average causal effect10 analysis	17
Harms	30	Sufficient detail on summarizing safety data, e.g., information on severity, expectedness, and causality;<br>details of how adverse events are coded or categorized; how adverse event data will be analysed, i.e., grade 3/4 only, incidence case analysis, intervention emergent analysis	18-19
Statistical software	31	Details of statistical packages to be used to carry out analyses	19
References	32a	References to be provided for nonstandard statistical methods	-
	32b	Reference to Data Management Plan	-
	32c	Reference to the Trial Master File and Statistical Master File	-
	32d	Reference to other standard operating procedures or documents to be adhered to	-

Taken from the paper: Gamble C, Krishan A, Stocken D, Lewis S, Juszczak E, Doré C, et al. Guidelines for the Content of Statistical Analysis Plans in Clinical Trials. JAMA. 2017;318(23):2337-43.

Abbreviations: CONSORT, Consolidated Standards of Reporting Trials; hbA1c, haemoglobin A1c; QoL, quality of life; SAP, statistical analysis plan.

For more information visit:

The development of this checklist was funded by the MRC Hubs for Trials Methodology Research
